# A short stereoselective synthesis of (+)-(6*R*,2′*S*)-cryptocaryalactone via ring-closing metathesis

**DOI:** 10.3762/bjoc.5.14

**Published:** 2009-04-24

**Authors:** Palakodety Radha Krishna, Krishnarao Lopinti, K L N Reddy

**Affiliations:** 1 D-206/B, Discovery Laboratory, Organic Chemistry Division-III, Indian Institute of Chemical Technology, Hyderabad-500 607, India, Fax: +91-40-27160387; 2 Government Degree College, Khairatabad, Hyderabad-500 004

**Keywords:** Carreira asymmetric alkynylation, *Cryptocarya bourdilloni* GAMB (Lauraceae), ring-closing metathesis, Sharpless asymmetric epoxidation

## Abstract

A short stereoselective synthesis of (+)-(6*R*,2′*S*)-cryptocaryalactone was successfully completed. Key steps included the application of Carreira’s asymmetric alkynylation reaction to form a propargylic alcohol and subsequently lactone formation using the powerful ring-closing metathesis reaction.

## Introduction

Natural products play an important role in the development of drugs and mankind has always taken advantage of nature as pharmacy: approximately 40% of the drugs that have been approved over the last years are either natural products or derivatives and analogs thereof [1–3]. Indeed, 5,6-dihydropyran-2-ones of both natural and non-natural origin have been found to be cytotoxic. In addition to many other relevant pharmacological properties [4–7], they inhibit HIV protease [8–9], induce apoptosis [10–15], and have even proved to be antileukemic [16]. At least some of these pharmacological effects may be related to the presence of the conjugated double bond, which acts as a Michael acceptor [17–23].

One of the sub-classes of these 5,6-dihydro-2*H*-pyran-2-one compounds is the styryl lactones which possess a styryl moiety side chain. The styryl moiety of goniothalamin has been shown to be of importance for its cytotoxic effect on different cancer cells as well as its antimicrobial, larvicidal activity and anti-inflammatory activity [24]. The styryl-pyrone skeleton is often found in natural products from Equisetaceae and also from the primitive angiosperm families, such as Lauraceae, Piperaceae, Ranunculaceae and Zingiberaceae.

**Figure 1:**
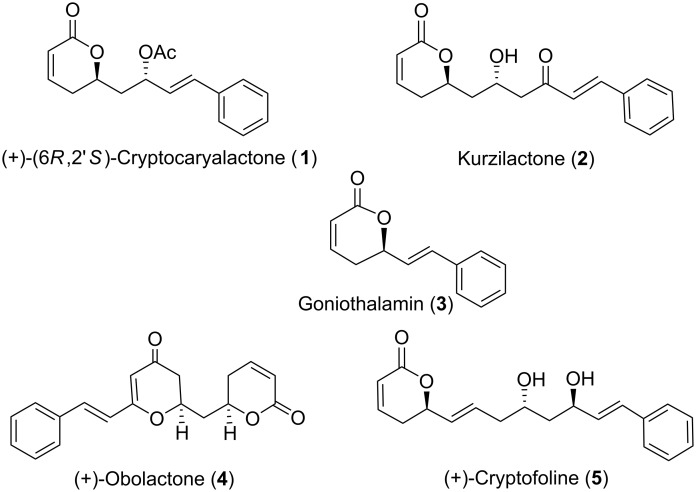
Some natural products containing styryl lactones.

Cryptocaryalactone **1** [25–26], kurzilactone (**2**) [27], goniothalamin (**3**) [28], (+)-obolactone (**4**) [29] and (+)-cryptofoline (**5**) [30] (Figure 1) are some of the naturally occurring styryl lactones. (+)-(6*R*,2′*S*)-Cryptocaryalactone (**1**) first featured in the phytochemical literature when its isolation from *Cryptocarya bourdilloni* GAMB (Lauraceae) was reported in 1972 by Govindachari [31–32]. Its absolute stereochemistry was established by H. H. Meyer through stereoselective synthesis [25]. Recently Yadav et al. have synthesized (6*R*,2′*S*)-cryptocaryalactone and its epimer using stereoselective reduction of δ-hydroxy-β-keto ester [33]. All other possible isomers of cryptocaryalactone were also isolated from *C. bourdilloni*, *C. moschata* and *C. myrtifolia* and their absolute configuration was established [34]. These cryptocaryalactones are natural germination inhibitors with no effect on corn [35]. We were interested in synthesizing natural products containing 5,6-dihydro-2*H*-pyran-2-one moiety [36–37], and herein we describe a short and efficient synthesis of cryptocaryalactone **1**.

## Results and Discussion

### Retrosynthetic analysis

**Figure 2:**
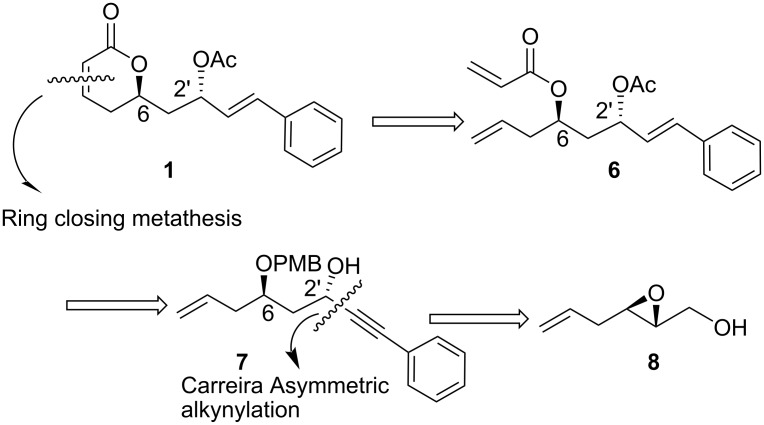
Retrosynthetic approach.

Retrosynthetic analysis (Figure 2) reveals that compound **1** could be synthesized from bis-olefin **6** by a ring-closing metathesis reaction, while the bis-olefin itself could be realised from the acryloylation of the corresponding homoallylic alcohol which in turn can be synthesized from **7**. Chiral propargyl alcohol **7** was obtained by the Carreira asymmetric alkynylation reaction of the corresponding aldehyde, which was synthesized from the corresponding primary alcohol that was obtained from a regioselective ring-opening reaction of 2,3-epoxy alcohol **8**.

**Scheme 1:**
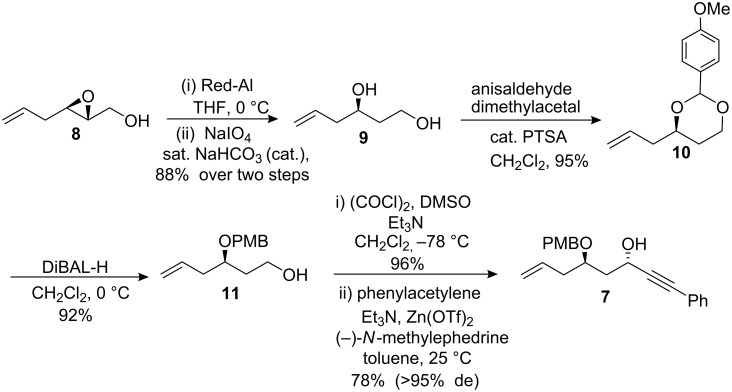
Synthesis of chiral propargyl secondary hydroxyl group.

The known 2,3-epoxy alcohol **8** was synthesized from the corresponding dienyl alcohol by the well-established Sharpless asymmetric epoxidation conditions in >94% *ee* as described in literature [38–39]. Compound **8** undergoes a reductive ring-opening reaction with Red-Al under standard reaction conditions to furnish the 1,3-diol **9** in 88% yield. Traces of 1,2-diol were oxidatively cleaved with NaIO_4_ in presence of catalytic amount of saturated NaHCO_3_ solution. Diol **9** was protected with anisaldehyde dimethylacetal in presence of PTSA to afford compound **10** in 95% yield, which was regioselectively opened with DIBAL-H to afford the primary alcohol **11** in 92% yield (Scheme 1).

The primary alcohol **11** was oxidized under Swern conditions and the crude aldehyde was exposed to the alkynylation reaction directly. Several base-mediated alkynylation conditions were examined to access the requisite propargyl alcohol **7** (Table 1).

**Table 1:** Asymmetric alkynylation with phenylacetylene.

Reagents	Solvent	Temperature	de^a^

*n*-BuLi	THF	−78 °C	28%
LDA	THF	−78 °C	56%
LDA/HMPA	THF	−78 °C	78%
Zn(OTf)_2_, Et_3_N, (−)-*N*-methylephedrine	toluene	25 °C	94%

^a^The diastereoselectivity was determined by NMR studies.

Amongst all of these, the Carreira asymmetric alkynylation gave excellent diastereomeric excess (>94% de) [40–41] and the propargyl alcohol **7** was obtained with the correct absolute configuration.

### Confirmation of absolute configuration

**Scheme 2:**
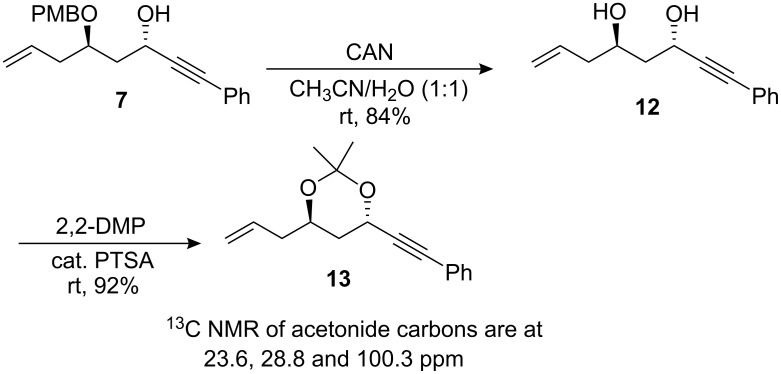
Determination of the stereochemistry of the 1,3-*anti* diols.

The structure of compound **7** was confirmed by ^1^H NMR and ^13^C spectral analysis (Scheme 2). The absolute stereochemistry was assigned based on Rychnovsky’s analogy [42–44].

According to literature precedent, the relative configuration of a secondary 1,3-diol can be assigned from the chemical shift of acetonide carbon atoms in ^13^C NMR spectrum. So, upon deprotection of **7**, diol **12** was obtained in good chemical yield (84%) and was further protected with 2,2-DMP, in presence of catalytic amount of PTSA, to furnish compound **13**. The analytical data of acetonide **13** confirmed the anti configuration of the 1,3-diol. Since the first hydroxyl center was obtained through an unambiguous method, the stereochemistry of the newly created hydroxyl functionality could be confirmed as that depicted in Scheme 2.

**Scheme 3:**
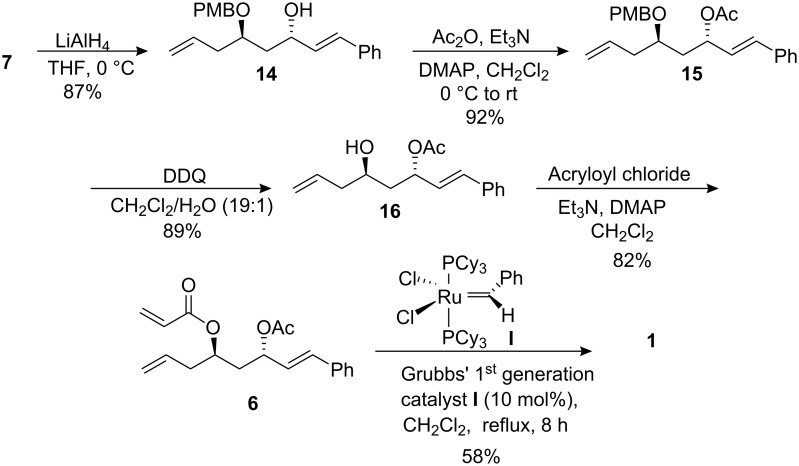
Synthesis of cryptocaryalactone by RCM.

The propargylic alcohol **7** was chemoselectively reduced with LiAlH_4_ in THF at 0 °C to give cinnamyl alcohol derivative **14** (87%, Scheme 3). Alcohol **14** was protected as its acetate under conventional reaction conditions. The PMB (*p*-methoxybenzyl protecting group) in compound **15** was selectively removed with DDQ in CH_2_Cl_2_/H_2_O (19:1) to afford homoallylic alcohol **16** (89%) without promoting the migration of the acetyl group. Finally, **16** was acrylated with acryloyl chloride/Et_3_N/CH_2_Cl_2_/0 °C to furnish the required bis-olefin **6** in 82% yield. Ring-closing metathesis of bis-olefin **6** with Grubbs’ 1^st^ generation catalyst (**I**; 10 mol%) [45] gave the required (+)-(6*R*,2′*S*)-cryptocaryalactone (**1**) as a solid in 58% yield {m.p. 122–125 °C /lit. 126–127 °C and [α]_D_= +20.1 (*c* = 0.20)/lit. +19.0 (*c* = 0.67)} [25]. All the spectral data matched with the literature values.

## Conclusion

In conclusion, a short stereoselective total synthesis of **1** has been accomplished by a convergent strategy wherein a chiral 2,3-epoxy alcohol was the starting material and Sharpless asymmetric epoxidation and Carreira asymmetric alkynylation were used as key steps for generating unambiguous assigned stereocenters. More importantly, the Grubbs’ ring-closing metathesis protocol was applied to construct the final 5,6-dihydropyrone ring of cryptocaryalactone. The advantage of this synthetic methodology is that one can in principle synthesize the other three diastereomers of cryptocaryalactone by altering the Sharpless epoxidation and Carreira’s conditions.

## Supporting Information

File 1 Experimental Data
