# Supplementary material for: A short stereoselective synthesis of (+)-(6R,2′S)-cryptocaryalactone via ring-closing metathesis
Source: Beilstein J Org Chem. 2009 Apr 24;5:14. doi: 10.3762/bjoc.5.14 (PMC2686312; doi:10.3762/bjoc.5.14)
Supplement: File 1 — Experimental Data [file Beilstein_J_Org_Chem-05-14-s001.doc]

Supporting information

A short stereoselective synthesis of (+)-(6*R*,2′*S*)-cryptocaryalactone via ring-closing metathesis

Palakodety Radha Krishna*^,1^, Krishnarao Lopinti^1^ and K. L. N. Reddy^2^

Address: ^1^D-206/B, Discovery Laboratory, Organic Chemistry Division-III, Indian Institute of Chemical Technology, Hyderabad-500 607, India, Fax: +91-40-27160387 and ^2^Government Degree College, Khairatabad, Hyderabad-500 004

Email: Palakodety Radha Krishna* - [prkgenius@iict.res.in](mailto:prkgenius@iict.res.in)

Corresponding author

Varian Gemini 200 MHz, Bruker Avance-300 MHz, Varian 400 MHz, 500 MHz, 600 MHz spectrometers with 7–10 mM solutions in deuteriated chloroform, tetramethylsilane as internal standard. *J* values are given in Hz. Optical rotations were measured with a JASCO P-1020 instrument and [α]_D_ values are in units of 10^−1^ deg cm^2^ g^−1^at 25 °C. IR spectra were taken with a Perkin–Elmer 1310 spectrometer. Mass spectra were recorded on CEC-21-11013 or Finnigan Mat 1210 double focusing mass spectrometers operating at a direct inlet system, FABMS and HRMS was measured using VG AUTOSPEC mass spectrometers at 5 or 7 k resolution using perflurokerosene as an internal reference. Elemental analysis was recorded on ELEMENTAR (Vario EL, Germany). Organic solutions were dried over anhydrous Na_2_SO_4_ and concentrated below 40 °C *in* *vacuo*. The software ACD/Name Version 1.0, developed by M/s Advanced Chemistry Development Inc., Toronto, Canada, assisted nomenclature used in the experimental section.

**(3*R*)-5-Hexene-1,3-diol (9)**

To a stirred solution of epoxide **8** (5 g, 43.85 mmol) in dry THF (50 mL) was added Red-Al (17.72 g, 87.72 mmol) at 0 °C. After 5 h the reaction mixture was quenched with sat. Na_2_SO_4_ solution (15 mL) and filtered through celite. The residue was washed with EtOAc (2 x 50 mL) then concentrated. To remove the traces of 1,2-diol formed, the crude residue was dissolved in dichloromethane and treated with NaIO_4_ (5.53 g, 25.86 mmol) in the presence of a catalytic amount of saturated NaHCO_3_ solution. The CH_2_Cl_2_ layer was filtered and dried over anhydrous Na_2_SO_4_ and concentrated. Crude product was purified by column chromatography (Silica gel 60–120, 35:65 EtOAc/*n*-hexane) to obtain 1,3-diol **9**.

Colorless liquid, yield: 4.42 g (88%); [α]_D_^25^ = −25.4 (*c* 3.20, CHCl_3_).

IR (neat): 3443, 2998, 2901, 1575, 923 cm^−1^.

^1^H NMR (200 MHz, CDCl_3_): δ 5.90–5.70 (m, 1H, olefin), 5.14–5.06 (m, 2H, olefin), 3.92–3.67 (m, 3H, OCH, OCH_2_), 3.35 (br. s, 2H, 2 x OH), 2.24 (t, *J* = 6.5 Hz, 2H, CH_2_), 1.75–1.57 (m, 2H, CH_2_).

ESIMS: 139 (M^+^+23).

Anal. Calcd for C_6_H_12_O_2_: C, 62.04; H, 10.41; Found: C, 62.11; H, 10.34.

**(4*R*)-4-Allyl-2-(4-methoxyphenyl)-1,3-dioxane (10)**

To a stirred solution of 1,3-diol **9** (4.2 g, 36.20 mmol) and anisaldehyde dimethylacetal (7.9 g, 43.44 mmol) in dry CH_2_Cl_2_ (45 mL) was added a catalytic amount of PTSA at 0 °C. After 1 h, the reaction mixture was quenched with Et_3_N (3 mL) and washed with water (2 x 15 mL), brine (2 x 15 mL), dried (Na_2_SO_4_) and concentrated under reduced pressure. The crude compound was purified by column chromatography (Silica gel 60–120, 5:95 EtOAc/*n*-hexane) to obtain 1,3-dioxane **10.**

Colorless liquid, yield: 8.04 g (95%); [α]_D_^25^ = +48.1 (*c* 2.30, CHCl_3_).

IR (neat): 2993, 2988, 2883, 1602, 1586, 856 cm^−1^.

^1^H NMR (300 MHz, CDCl_3_): δ 7.33 (d, *J* = 8.3 Hz, 2H, Ar-H), 6.80 (d, *J* = 8.3 Hz, 2H, Ar-H), 5.90–5.77 (m, 1H, olefin), 5.39 (s, 1H, Ar-CH), 5.12–5.04 (m, 2H, olefin), 4.20 (dd, *J* = 5.3, 11.3 Hz, 1H, OCH), 3.87 (dd, *J* = 3.0, 11.3 Hz, 1H, OCH), 3.85–3.3.77 (m, 1H, OCH), 3.77 (s, 3H, OCH_3_), 2.38 (qt, *J* = 6.0, 13.6, 20.4 Hz, 2H, CH_2_), 1.75 (ddd, *J* = 5.3, 12.8 Hz, 1H, CH), 1.50 (d, *J* = 12.8 Hz, 1H, CH).

ESIMS: 235 (M^+^+H).

Anal. Calcd for C_14_H_18_O_3_: C, 71.77; H, 7.74; Found: C, 71.73; H, 7.78.

**(3*R*)-3-[(4-Methoxybenzyl)oxy]-5-hexen-1-ol (11)**

DIBAL-H (45.5 mL, 32.05 mmol, 10% solution in toluene) was added to a stirred solution of **10** (5.0 g, 21.73 mmol) in dry CH_2_Cl_2_ (50 mL) at 0 °C drop wise and stirred for 30 min. After the completion of the reaction methanol (15 mL) was added, followed by saturated sodium potassium tartrate solution (15 mL), filtered and washed with EtOAc (2 x 100 mL). The organic layer was dried over anhydrous Na_2_SO_4_ and concentrated under reduced pressure and the residue was subjected to column chromatography (60–120 Silica gel, 20:80 EtOAc/*n*-hexane) to obtain **11.**

Light yellow syrup, yield: 4.63 g (92%); [α]_D_^25^ = −69.8 (*c* 1.45, CHCl_3_).

IR (neat): 3432, 2982, 2892, 1613, 1545, 923 cm^−1^.

^1^H NMR (300 MHz, CDCl_3_): δ 7.19 (d, *J* = 8.3 Hz, 2H, Ar-H), 6.80 (d, *J* = 8.3 Hz, 2H, Ar-H), 5.83–5.70 (m, 1H, olefin), 5.10–5.04 (m, 2H, olefin), 4.47 (q, *J* = 10.6, 52.0 Hz, 2H, Ar-CH_2_), 3.77 (s, 3H, OCH_3_), 3.73–3.58 (m, 3H, OCH, OCH_2_), 2.43–2.24 (m, 2H, CH_2_), 2.21 (br. s, 1H, OH), 1.72–1.66 (m, 2H, CH_2_).

ESIMS: 237 (M^+^+H).

Anal. Calcd for C_14_H_20_O_3_: C, 71.16; H, 8.53; Found: C, 71.13; H, 8.54.

**(3*S*,5*R*)-5-[(4-Methoxybenzyl)oxy]-1-phenyl-7-octen-1-yn-3-ol (7)**

To a stirred solution of oxaloyl chloride (0.36 g, 2.80 mmol) and DMSO (0.44 g, 5.60 mmol) in CH_2_Cl_2_ (5 mL) was added alcohol **11** (0.60 g, 2.54 mmol) at −78 °C. After 1 h, triethylamine (1.54 g, 15.24 mmol) was added and stirred for another 1 h at room temperature. The organic layer was washed with water (2 x 10 mL) and brine (2 x 10 mL) solution and dried over anhydrous Na_2_SO_4_ and concentrated. The crude aldehyde was used as such in the next step.

(−)-*N*-methylephedrine (1.00 g, 5.64 mmol) and zinc triflate (1.86 g, 5.12 mmol) were suspended under N_2_ in dry toluene (5 mL), followed by addition of triethylamine (0.57 g, 5.64 mmol). The resulting mixture was stirred at room temperature for 2 h. After addition of phenylacetylene (0.58 g, 5.64 mmol), the stirring was continued for 30 min. A solution of aldehyde (0.6 g, 2.56 mmol) in dry toluene (5 mL) was then added via syringe. The reaction mixture was stirred at room temperature for 5 h. The reaction mixture was washed with water (2 x 10 mL) and sat. NH_4_Cl solution (2 x 10 mL) and extracted in to ether (2 x 20 mL). The organic layer was dried over anhydrous Na_2_SO_4_ and concentrated under vacuum pressure. The crude residue was purified by column chromatography (60–120 Silica gel,15:85 EtOAc/*n*-hexane) to obtain **7.**

Colorless liquid, yield: 0.67 g, 78%); [α]_D_^25^ = −182.3 (*c* 2.75, CHCl_3_).

IR (neat): 3405, 2987, 2890, 2206, 1632, 1505, 876 cm^−1^.

^1^H NMR (200 MHz, CDCl_3_): δ 7.39–7.28 (m, 2H, Ar-H), 7.27–7.24 (m, 3H, Ar-H), 7.20 (d, *J* = 9.2 Hz, 2H, Ar-H), 6.83 (d, *J* = 9.2 Hz, 2H, Ar-H), 5.94–5.67 (m, 1H, olefin), 5.15–5.07 (m, 2H, olefin), 4.72 (t, *J* = 6.6 Hz, 1H, OCH), 4.50 (q, *J* = 11.8, 40.7 Hz, 2H, Ar-CH_2_), 3.80–3.69 (m, 1H, OCH), 3.76 (s, 3H, OCH_3_), 2.84 (br. s, 1H, OH), 2.40 (t, *J* = 6.6 Hz, 2H, CH_2_), 2.16–1.83 (m, 2H, CH_2_).

^13^C NMR (75 MHz, CDCl_3_): δ 134.1, 131.9, 129.4, 128.2, 118.2, 113.8, 89.9, 84.5, 77.5, 70.3, 61.4, 54.5, 42.2, 38.1.

ESIMS: 337 (M^+^+H).

Anal. Calcd for C_22_H_24_O_3_: C, 78.54; H, 7.19; Found: C, 78.58; H, 7.20.

**(3*S*,5*R*)-1-Phenyl-7-octen-1-yne-3,5-diol (12)**

To a stirred solution of propargyl alcohol **7** (0.20 g, 0.59 mmol) in CH_3_CN/H_2_O (1:1; 5 mL) was added ceric ammonium nitrate (0.49 g, 0.89 mmol) at room temperature. After stirring for 1 h, the reaction mixture was quenched with saturated NaHCO_3_ solution. The reaction mixture was extracted with EtOAc (2 x 10 mL) and washed with water (2 x 5 mL) and brine (2 x 5 mL). The organic layer was dried over anhydrous Na_2_SO_4_, concentrated and purified by column chromatography (Silica gel, 60–120 mesh, EtOAc/*n*-hexane, 20:80) to obtain diol **12.**

A white solid with mp 76–78 °C, yield: 0.108 g (84%); [α]_D_^25^ = +27.33 (*c* 0.15, CHCl_3_).

IR (neat): 3447, 2957, 2214, 1516, 784 cm^−1^.

^1^H NMR (200 MHz, CDCl_3_): δ 7.43–7.36 (m, 2H, Ar-H), 7.32–7.23 (m, 3H, Ar-H), 5.95–5.72 (m, 1H, olefin), 5.23–5.07 (m, 2H, olefin), 4.83 (t, *J* = 6.3 Hz, 1H, OCH), 3.97 (p, *J* = 6.3, 12.5 Hz, 1H, OCH), 2.70 (br. s, 2H, 2 x OH), 2.29 (t, *J* = 6.3 Hz, 2H, CH_2_), 1.96 (t, *J* = 6.3 Hz, 2H, CH_2_).

ESIMS: 239 (M^+^+23).

Anal. Calcd for C_14_H_16_O_2_: C, 77.75; H, 7.46; Found: C, 77.72; H, 7.48.

**(4*R*,6*S*)-4-Allyl-2,2-dimethyl-6-(2-phenyl-1-ethynyl)-1,3-dioxane (13)**

To a stirred solution of diol **12** (0.07 g, 0.32 mmol) in 2,2-dimethoxypropane (2 mL) added a catalytic amount of PTSA at room temperature. After stirring for 1 h, the reaction mixture was quenched with Et_3_N (0.5 mL) and concentrated. The crude residue was purified by column chromatography (Silica gel, 60–120 mesh, EtOAc/*n*-hexane, 05:95) to afford acetonide **13.**

Colorless liquid, yield: 0.076 g (92%); [α]_D_^25^ = −33.29 (*c* 0.45, CHCl_3_).

IR (neat): 2952, 2900, 2213, 1514, 782 cm^−1^.

^1^H NMR (200 MHz, CDCl_3_): δ 7.45–7.35 (m, 2H, Ar-H), 7.31–7.23 (m, 3H, Ar-H), 5.90–5.70 (m, 1H, olefin), 5.14–5.04 (m, 2H, olefin), 4.91 (t, *J* = 5.2 Hz, 1H, OCH), 4.17 (p, *J* = 5.9, 12.2 Hz, 1H, OCH), 2.40–2.12 (m, 2H, CH_2_), 1.91–1.82 (m, 2H, CH_2_), 1.42 (s, 3H, CH_3_), 1.39 (s, 3H, CH_3_).

^13^C NMR (75 MHz, CDCl_3_): δ 133.9, 131.5, 128.4, 128.3, 122.7, 117.2, 100.3, 89.5, 85.6, 65.4, 60.1, 40.2, 36.6, 28.8, 23.6.

ESIMS: 279 (M^+^+23).

Anal. Calcd for C_17_H_20_O_2_: C, 79.65; H, 7.86; Found: C, 79.66; H, 7.84.

**(1*E*,3*S*,5*R*)-5-[(4-Methoxybenzyl)oxy]-1-phenyl-1,7-octadien-3-ol (14)**

To a stirred solution of LiAlH_4_ (0.066 g, 1.78 mmol) in dry THF (2 mL) was added **7** (0.47 g, 1.39 mmol) dissolved in THF (5 mL) at 0 °C. After stirring for 1 h, the reaction mixture was quenched with saturated Na_2_SO_4_ solution at 0 °C then filtered. The residue was washed with EtOAc (3 x 10 mL) and the EtOAc layer was concentrated under reduced pressure. The crude product was purified by column chromatography (60–120 Silica gel, 15:85 EtOAc/*n*-hexane) to obtain **14**.

Colorless liquid, yield: 0.41 g (87%); [α]_D_^25^ = −86.0 (*c* 0.50, CHCl_3_).

IR (neat): 3417, 2982, 2887, 1626, 1512, 974, 723 cm^−1^.

^1^H NMR (200 MHz, CDCl_3_): δ 7.32–7.16 (m, 7H, Ar-H), 6.86 (d, *J* = 8.4 Hz, 2H, Ar-H), 6.50 (d, *J* = 15.8 Hz, 1H, olefin), 6.11 (dd, *J* = 6.2, 16.2 Hz, olefin), 5.89–5.68 (m, 1H, olefin), 5.13–5.05 (m, 2H, olefin), 4.51 (q, *J* = 11.0, 50.7 Hz, 2H, PhCH_2_), 4.43–4.36 (m, 1H, OCH), 3.79 (s, 1H, OCH_3_), 3.78–3.64 (m, 1H, OCH), 3.34 (s, 1H, OH), 2.38 (t, *J* = 6.6 Hz, 2H, CH_2_), 1.84–1.66 (m, 2H, CH_2_).

ESIMS: 361 (M^+^+23).

Anal. Calcd for C_22_H_26_O_3_: C, 78.07; H, 7.74; Found: C, 78.04; H, 7.76.

**(1*S*,3*R*)-3-[(4-Methoxybenzyl)oxy]-1-[(*E*)-2-phenyl-1-ethenyl]-5-hexenyl acetate (15)**

To a stirred solution of **14** (0.10 g, 0.29 mmol) in CH_2_Cl_2_ (3 mL) was added triethylamine (0.15 g, 1.47 mmol) and Ac_2_O (0.05 mL, 0.44 mmol) in the presence of a catalytic amount of DMAP and stirred for 3 h. The reaction mixture was diluted with CH_2_Cl_2_ (5 mL), washed with water (2 x 5 mL), brine (2 x 5 mL), dried (Na_2_SO_4_) and concentrated under reduced pressure. The crude product was purified by column chromatography (60–120 Silica gel, 08:92 EtOAc/*n*-hexane) to obtain **15.**

Colorless liquid, yield: 0.103 g (92%); [α]_D_^25^ = −47.6 (*c* 1.10, CHCl_3_).

IR (neat): 2986, 2885, 1753, 1630, 1523, 914 cm^−1^.

^1^H NMR (200 MHz, CDCl_3_): δ 7.27–7.19 (m, 7H, Ar-H), 6.82 (d, *J* = 8.6 Hz, 2H, Ar-H), 6.41 (d, *J* = 16.0 Hz, 1H, olefin), 5.96 (dd, *J* = 7.8, 16.0 Hz, 1H, olefin), 5.91–5.69 (m, 1H, olefin), 5.48 (q, *J* = 7.4, 13.7 Hz, 1H, OCH), 5.12–5.04 (m, 2H, olefin), 4.41 (q, *J* = 11.3, 42.6 Hz, 2H, PhCH_2_), 3.77 (s, 3H, OCH_3_), 3.52–3.41 (m, 1H, OCH), 2.34 (t, *J* = 6.6 Hz, 2H, CH_2_), 2.00 (s, 3H, COOCH_3_), 2.06–1.92 (m, 1H, CH), 1.83–1.70 (m, 1H, CH).

ESIMS: 403 (M^+^+23).

Anal. Calcd for C_24_H_28_O_4_: C, 75.76; H, 7.42; Found: C, 75.71; H, 7.44.

**(1*S*,3*R*)-3-Hydroxy-1-[(*E*)-2-phenyl-1-ethenyl]-5-hexenyl acetate (16)**

To a stirred solution of **15** (0.10 g, 0.26 mmol) in CH_2_Cl_2_/H_2_O (19:1) (3 mL) was added DDQ (0.07 g, 0.32 mmol) at 0 °C and the reaction mixture stirred at room temperature for 30 min. Then saturated NaHCO_3_ (1 mL) was added and extracted with CH_2_Cl_2_ (2 x 5 mL), the combined organic layers were washed with water (2 x 5 mL), brine (2 x 5 mL), dried over anhydrous Na_2_SO_4_ and concentrated. The crude residue was purified by column chromatography (60–120 Silica gel, 10:90 EtOAc/*n*-Hexane) to afford **16**.

Colorless liquid, yield: 0.057 g (89%); [α]_D_^25^ = +29.3 (*c* 0.70, CHCl_3_).

IR (neat): 3422, 2943, 2881, 1750, 1621, 1516, 912 cm^−1^.

^1^H NMR (200 MHz, CDCl_3_): δ 7.38–7.20 (m, 5H, Ar-H), 6.64 (d, *J* = 16.0 Hz, 1H, olefin), 6.07 (dd, *J* = 7.8, 16.0 Hz, 1H, olefin), 5.89–5.68 (m, 1H, olefin), 5.56 (q, *J* = 7.0, 14.1 Hz, 1H, OCH), 5.16–5.04 (m, 2H, olefin), 3.77–3.67 (m, 1H, OCH), 2.42–2.14 (m, 2H, CH_2_), 2.06 (s, 3H, COOCH_3_), 1.99–1.72 (m, 2H, CH_2_).

^13^C NMR (75 MHz, CDCl_3_): δ 134.1, 133.1, 128.6, 128.0, 127.1, 126.6, 118.6, 73.0, 67.8, 42.2, 41.4, 21.4.

ESIMS: 283 (M^+^+23).

Anal. Calcd for C_16_H_20_O_3_: C, 73.82; H, 7.74; Found: C, 73.79; H, 7.77.

**(1*R*,3*S*,4*E*)-3-Acetoxy-1-allyl-5-phenyl-4-pentenyl acrylate (6)**

To a stirred solution of **16** (0.05 g, 0.20 mmol) in CH_2_Cl_2_ was added triethylamine (0.062 g, 0.61 mmol) and acryloyl chloride (0.028 g, 0.31 mmol) in the presence of a catalytic amount of DMAP (*N*,*N*-dimethylaminopyridine) and stirred for 2 h. The reaction mixture was diluted with CH_2_Cl_2_ (5 mL), washed with water (2 x 5 mL), brine (2 x 5 mL), dried over anhydrous Na_2_SO_4_ and concentrated under reduced pressure. The crude product was purified by column chromatography (60–120 Silica gel, 5:95 EtOAc/*n*-hexane) to obtain **6.**

Colorless liquid, yield: 0.052 g (82%); [α]_D_^25^ = +39.8 (*c* 0.60, CHCl_3_).

IR (neat): 2984, 2881, 1746, 1698, 1602, 1502, 946 cm^−1^.

^1^H NMR (200 MHz, CDCl_3_): δ 7.37–7.23 (m, 5H, Ar-H), 6.57 (d, *J* = 14.7 Hz, 1H, olefin), 6.42 (dd, *J* = 2.67, 17.4 Hz, 1H, olefin), 6.18–6.01 (m, 2H, olefin), 5.89–5.66 (m, 2H, olefin), 5.44 (q, *J* = 6.7, 14.7 Hz, 1H, OCH), 5.16–5.02 (m, 3H, olefin, OCH), 2.42 (t, *J* = 5.3 Hz, 2H, CH_2_), 2.09 (s, 3H, COOCH_3_), 2.26–1.88 (m, 2H, CH_2_).

ESIMS: 337 (M^+^+23).

Anal. Calcd for C_19_H_22_O_4_: C, 72.59; H, 7.05; Found: C, 72.57; H, 7.01.

**(+)-(6*R*,2′*S*)-Cryptocaryalactone**

Bis-olefin **6** (0.05 g, 0.16 mmol) dissolved in freshly distilled degassed anhydrous CH_2_Cl_2_ (30 mL), was treated with Grubbs' catalyst **I** (0.013 g, 0.014 mmol) and heated at reflux for 8 h under N_2_ atmosphere. Most of the solvent was then distilled off and the concentrated solution left to stir at room temperature for 2 h under air bubbling in order to decompose the catalyst. The reaction mixture was evaporated to dryness to give a brown residue, which was purified by column chromatography (Silica gel, 60–120 mesh, EtOAc/*n*-hexane, 45:55) to afford **1.**

A solid with mp 122–125 °C, yield: 0.022 g (58%); [α]_D_^25^ = +20.1 (*c* 0.20, CHCl_3_).

IR (neat): 2986, 2887, 1713, 1693, 784 cm^−1^.

^1^H NMR (200 MHz, CDCl_3_): δ 7.37–7.16 (m, 5H, Ar-H), 6.85–6.76 (m, 1H, olefin), 6.63 (d, *J* = 16.0 Hz, 1H, olefin), 6.07 (dd, *J* = 7.8, 16.0 Hz, olefin), 5.96 (dt, *J* = 2.0, 9.8 Hz, 1H, olefin), 5.60 (q, *J* = 7.0 Hz, 1H, CHCO), 4.46 (dq, *J* = 5.1, 9.6 Hz, 1H, OCH), 2.40–2.21 (m, 2H, CH_2_), 2.02 (s, 3H, COCH_3_), 2.06–1.90 (m, 2H, CH_2_).

^13^C NMR (75 MHz, CDCl_3_): δ 160.9, 144.4, 133.7, 131.9, 128.6, 128.3, 126.7, 126.1, 121.5, 74.7, 71.2, 39.8, 29.7, 29.4, 21.2.

ESIMS: 309 (M^+^+23).

HRMS(ESI): *m/z* calcd for C_48_H_56_O_6_Na: 309.1102; Found: 309.1112.
